# Associations between smoking, sex hormone levels and late-onset hypogonadism in men differ depending on age

**DOI:** 10.18632/aging.202442

**Published:** 2021-02-01

**Authors:** Qian Liu, Xiangchi Peng, Yiqun Gu, Xuejun Shang, Yuanzhong Zhou, Huiping Zhang, Liandong Zuo, Guangan Mei, Chengliang Xiong, Honggang Li, Xiangbin Kong

**Affiliations:** 1 Center for Reproductive Medicine, Wuhan Children’s Hospital, Tongji Medical College, Huazhong University of Science and Technology, Wuhan 430000, China; 2 Institute of Reproductive Health, Tongji Medical College, Huazhong University of Science and Technology, Wuhan 430030, China; 3 National Health and Family Planning Key Laboratory of Male Reproductive Health, National Research Institute for Family Planning, Beijing 100000, China; 4 Department of Andrology, Jinling Hospital, School of Medicine, Nanjing University, Nanjing 210093, China; 5 School of Public Health, Zunyi Medical University, Zunyi 563000, Guizhou, China; 6 Wuhan Tongji Reproductive Medicine Hospital, Wuhan 563000, China; 7 Guangzhou Women and Children’s Medical Center, Guangzhou 510000, China; 8 Technical Guidance Institute of Shanxi Province Family Planning Commission, Xi’an 710000, China; 9 Department of Reproductive Medicine, First Affiliated Hospital of Wenzhou Medical University, Wenzhou 325000, China

**Keywords:** aging male’s symptom scale, cigarette smoking, late-onset hypogonadism, sex hormone-binding globulin, sex hormones

## Abstract

Few studies have investigated whether associations between smoking, sex hormone levels, and symptoms of late-onset hypogonadism (LOH) in men are affected by age. This multi-center, cross-sectional study involving 6,296 men aged 40-79 years was conducted between June 1, 2013 and August 31, 2016 in 6 provinces of China. Total testosterone, free testosterone, and Aging Males’ Symptoms scale (AMS) scores were compared depending on smoking status and the number of cigarettes smoked. Total testosterone was higher in smokers than in non-smokers in all except the 70-79 year old subgroup. Free testosterone was higher in smokers than non-smokers for the 40-49 and 50-59 year old subgroups, but not the 60-69 and 70-79 year old subgroups. Total testosterone was positively associated with number of cigarettes consumed in smokers aged 40-49 and 50-59 years. Sexual and somatic AMS scores were higher in current and ex-smokers than in non-smokers in all age subgroups from 40 to 79 years and were negatively associated with cigarette consumption in smokers aged 40-49 years. These results indicate that, as men age, the positive association between smoking and testosterone weakens, while the positive association between smoking and LOH symptoms becomes stronger.

## INTRODUCTION

Testicular testosterone secretion in men over 40 years of age decreases by 1-2% per year [1], and some middle-aged and elderly men develop testosterone deficiency, which is associated with diffuse symptoms including sexual dysfunction, muscle weakness, osteoporosis, hot flashes, insomnia, fatigue, depression, irritability, and anxiety [1, 2]. Late-onset hypogonadism (LOH) involves a cluster of these symptoms together with testosterone deficiency [1–4], and LOH symptoms are widely assessed using the Aging Males’ Symptoms (AMS) scale, which consists of 17 items organized in sexual, somatic, and psychological domains (https://www.aging-males-symptoms-scale.info/languages.htm) [5–7].

Some LOH symptoms have been linked not only to aging but also to cigarette smoking [8–10]; these include erectile dysfunction [11, 12], sarcopenia [13, 14], frailty [15], depression [16], anxiety [17]. and sleep disorder [18, 19]. Cigarette smoking, in turn, has been linked to changes in serum levels of sex hormones [20–27]. While many studies have reported higher serum concentrations of total testosterone (TT) and sex hormone-binding globulin (SHBG) in current smokers than in those who never smoked [20, 21, 27], others found no differences between those groups [22–24]. Similarly, some, but not all, studies have associated smoking with free testosterone and luteinizing hormone (LH) levels [20, 21, 25, 26]. The underlying causes of these discrepancies remain unclear, but differences between the patient groups in different studies, e.g., participants of different age profiles, might be at least partially responsible [20–27].

In this study, we explored whether age-dependent heterogeneity exists in associations between smoking, sex hormone levels, and LOH symptoms in men. We also simultaneously evaluated associations between cigarette smoking, sex hormone levels, and LOH symptoms in contrast to most previous studies, which focused only on sex hormones or on LOH symptoms. Our goal was to improve the understanding of associations between smoking and reproductive health in men. In addition, we attempted to determine whether smoking may contribute to difficulty in diagnosing LOH, which is complicated by the fact that relatively few men older than 40 years simultaneously present both testosterone deficiency and LOH symptoms [1, 2, 4, 28].

## RESULTS

### Participant characteristics

A total of 6,296 participants were recruited from the following provinces: Jiangsu (representing the eastern part of the country), n = 966; Guizhou (southwestern), n = 921; Shanxi (northeastern), n = 1,127; Hebei (northern), n = 1,093; Guangdong (southern), n = 1,038; and Hubei (central), n = 1,151. The final analysis included 5,965 men after excluding 217 who did not fill out questionnaires, 15 who did not provide information on smoking exposure, and 99 who had hypothalamus-pituitary-testis axis disease or who were taking medications that could affect testosterone levels.

Characteristics of the study population are shown in Table 1. The mean age was 55.93 ± 0.12 years, and the mean BMI was 24.23 ± 0.04 kg/m2. The age distribution was as follows: 1,789 (30.0%) were 40-49 years old; 1,977 (33.1%), 50-59; 1,685 (28.2%), 60-69; and 514 (8.6%), 70-79. The distribution of smoking status was as follows: 1,884 (31.6%) were never smokers, 3,389 (56.8%) were current smokers, and 692 (11.6%) were past smokers. Among the 3,389 current smokers, 747 (22.0%) smoked < 10 cigarettes/day, 1,300 (38.4%) smoked 10-20 cigarettes/day, and 1,342 (39.6%) smoked > 20 cigarettes/day. 100 current smokers (3.0%) had smoked for 1-5 years, 154 (4.5%) for 6-10 years, 207 (6.1%) for 11-15 years, and 2,928 (86.4%) for ≥16 years. After adjusting for age, number of cigarettes per day, BMI, and alcohol intake, smoking years was not associated with sex hormones levels or AMS scores (p>0.05 for all).

**Table 1:** Characteristics of the study population.

**Characteristic**	**Total n = 5,965**	**Smoking status**			
		**Never-smokers n=1884**	**Current smokers n=3389**	**Past smokers n=692**	***P* value**
**Age, yr**	55.93 ± 0.12	55.65 ± 0.23a	55.66 ± 0.16a	58.04 ± 0.35b	< 0.001
**Age subgroups, yr**					
40-49	1789 (30.0)	618 (32.8)	1030 (30.4)	141 (20.4)	
50-59	1977 (33.1)	576 (30.6)	1161 (34.3)	240 (34.7)	< 0.001
60-69	1685 (28.2)	506 (26.8)	949 (28.0)	230 (33.2)	
70-79	514 (8.6)	184 (9.8)	249 (7.3)	81 (11.7)	
**BMI, kg/m^2^**	24.23 ± 0.04	24.71 ± 0.08a	23.93 ± 0.06b	24.38 ± 0.12c	< 0.001
**BMI subgroups, kg/m^2^**					
≤ 23.9	2929 (49.1)	820 (43.5)	1794 (53.0)	315 (45.5)	
24.0-27.9	2233 (37.4)	736 (39.1)	1197 (35.3)	300 (43.4)	< 0.001
≥ 28.0	803 (13.5)	328 (17.4)	398 (11.7)	77 (11.1)	
**Cigarettes/day**					
<10	—	—	747 (22.0)	—	—
10-20	—	—	1300 (38.4)	—	—
>20	—	—	1342 (39.6)	—	—
**Years of smoking, yr**				—	
1-5			100 (3.0)		
6-10			154 (4.5)		
11-15			207 (6.1)		
≥16			2928 (86.4)		
**Alcohol intake**					
Never	1664 (27.9)	730 (38.7)	793 (23.4)	141 (20.4)	
Occasionally	2188 (36.7)	730 (38.7)	1217 (35.9)	241 (34.8)	< 0.001
Frequently	1798 (30.1)	390 (20.8)	1198 (35.3)	210 (30.3)	
Quit	315 (5.3)	34 (1.8)	181 (5.3)	100 (14.5)	

Data are presented as mean ± standard error or as n (%). ^a,b,c^ Values in the same row marked with different letters differ significantly from each other.

### Associations between cigarette smoking and sex hormones

### Smoking status

**Figure 1:**
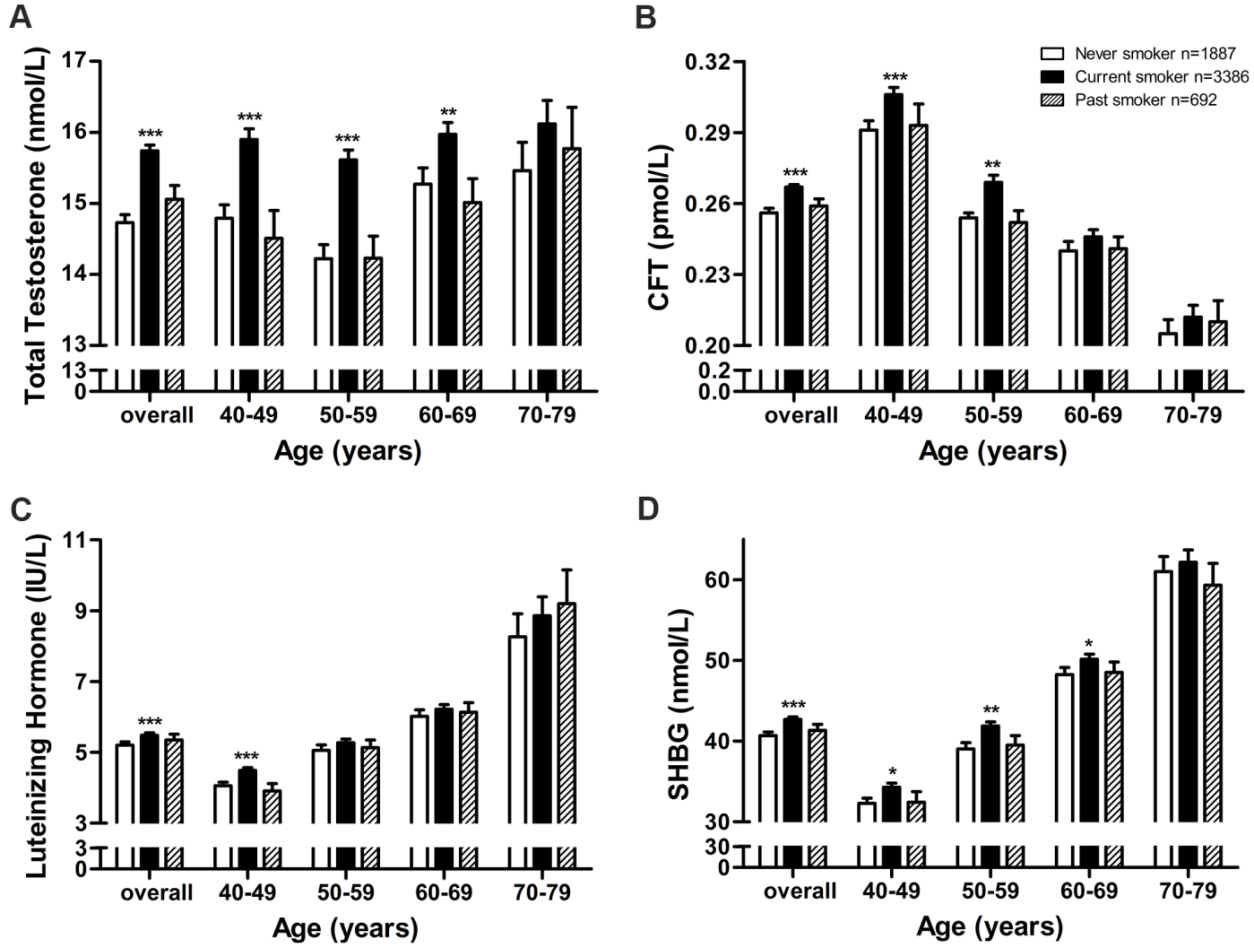
**Adjusted mean sex hormone levels by smoking status in middle-aged and elderly men.** Adjusted mean levels of (**A**) total testosterone, (**B**) calculated free testosterone, (**C**) luteinizing hormone, and (**D**) sex hormone-binding globulin by smoking status in middle-aged and elderly men. CFT, calculated free testosterone; SHBG, sex hormone-binding globulin. Geometric mean values were calculated using multiple covariance and adjusting for age (in “overall” analysis only), body mass index, and alcohol intake. Error bars indicate standard error. *P < 0.05, ** P < 0.01, *** P < 0.001 vs. never smokers.

Serum TT was significantly higher in current smokers (15.74 nM) than in past smokers (15.06 nM, P < 0.004) and never smokers (14.73 nM, P < 0.001; Figure 1A), and this relationship was observed in all but the 70-79 age subgroup. TT was similar between past smokers and never smokers (P = 0.162, Figure 1A), and this result was obtained in all four age subgroups.

Similarly, serum CFT was significantly higher in current smokers (0.267 pM) than in past smokers (0.259 pM, P = 0.010) and never smokers (0.256 pM, P < 0.001, Figure 1B), and this relationship was observed in the two younger age subgroups but not the two older subgroups. CFT was similar between past smokers and never smokers (P = 0.364, Figure 1B), and this result was obtained in all four age subgroups.

Serum LH was significantly higher in current smokers than never smokers (5.48 vs. 5.20 IU/L, P < 0.001, Figure 1C), and this relationship was observed in the 40-49 age subgroup but not in older subgroups. LH was similar between past smokers and never smokers (5.35 vs. 5.20 IU/L, P = 0.204, Figure 1C), and this result was obtained in all four age subgroups.

Serum SHBG was significantly higher in current smokers (42.69 nM) than in past smokers (41.35 nM, P = 0.047) and never smokers (40.69 nM, P < 0.001, Figure 1D), and this relationship was observed in all except the oldest subgroup. SHBG was similar between past smokers and never smokers (P = 0.370, Figure 1D), and this result was obtained in all four age subgroups.

### Number of cigarettes per day

**Figure 2:**
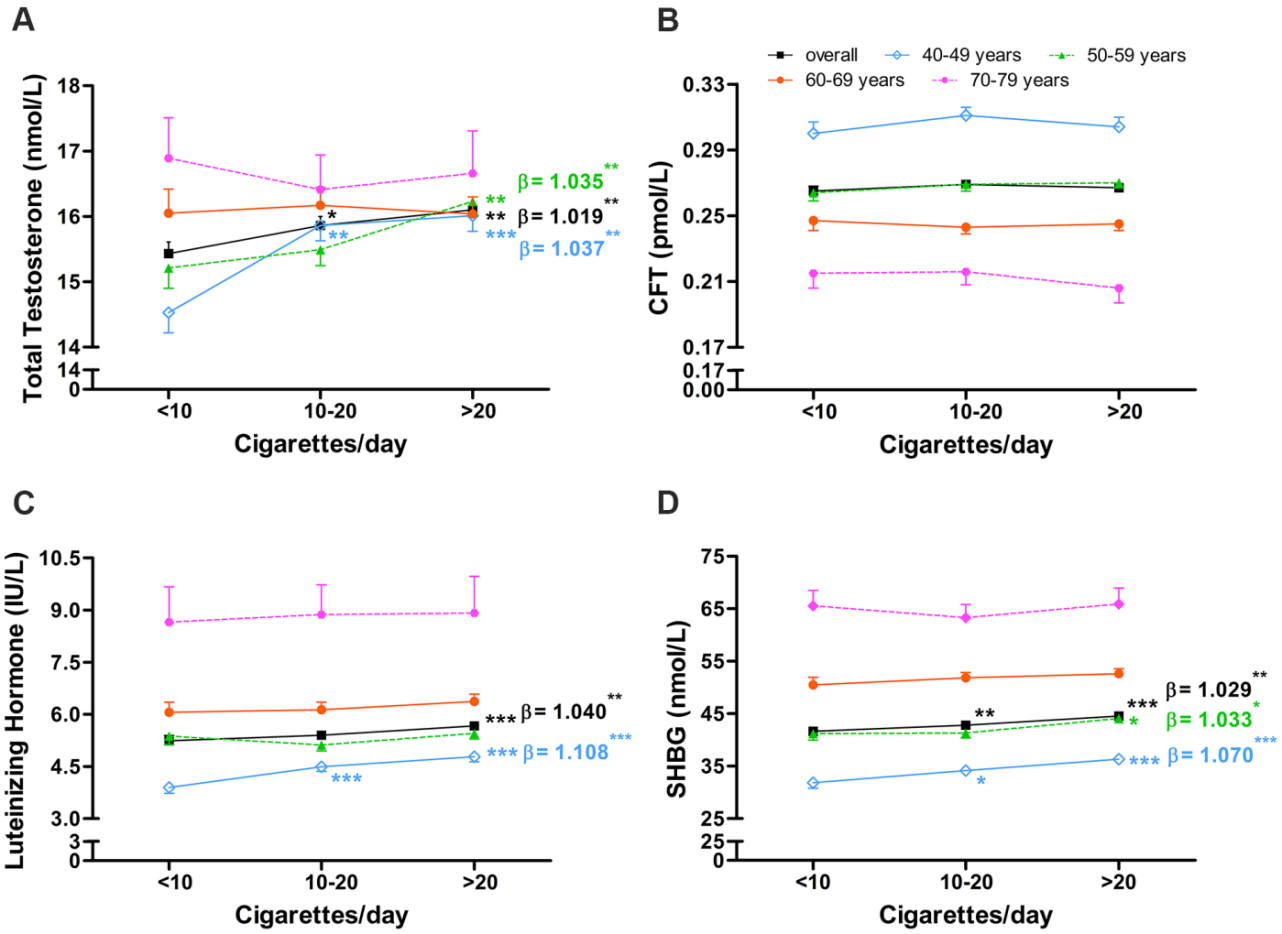
**Association between number of cigarettes smoked per day and serum sex hormone levels in middle-aged and elderly men.** Association between number of cigarettes smoked per day and serum levels of (**A**) total testosterone, (**B**) calculated free testosterone, (**C**) luteinizing hormone, and (**D**) sex hormone-binding globulin in middle-aged and elderly men. CFT, calculated free testosterone; SHBG, sex hormone-binding globulin. Geometric mean values were calculated using multiple covariance and adjusting for age (in “overall” analysis only), body mass index, and alcohol intake. Error bars indicate standard error. *P < 0.05, ** P < 0.01, *** P < 0.001 vs. current smokers who smoked <10 cigarettes/day. Regression coefficients were obtained from multiple regression adjusting for age (in “overall” analysis only), body mass index, and alcohol intake and were calculated separately for daily cigarette consumption subgroups, which were ordinally categorized as <10, 10-20, >20 cigarettes/day. *P < 0.05, ** P < 0.01 and *** P < 0.001 for regression coefficients.

Among current smokers, TT was significantly higher among those who smoked > 20 cigarettes/day (16.10 nM, P = 0.002) or 10-20 cigarettes/day (15.86 nM, P = 0.042) than among those who smoked < 10 cigarettes/day (15.43 nM, Figure 2A). TT remained positively associated with daily cigarette consumption after adjusting for age, BMI, and alcohol intake (α = 1.019, P = 0.003, Figure 2A). This positive association was observed in the two younger age subgroups, but not in the two older subgroups. CFT did not differ significantly depending on daily cigarette consumption (Figure 2B), and this lack of association was observed in all four age subgroups.

LH was significantly higher among current smokers who smoked > 20 cigarettes/day (5.67 IU/L) than among those who smoked 10-20 (5.40 IU/L, P = 0.022) or <10 cigarettes/day (5.24 IU/L, P = 0.002, Figure 2C). LH was positively associated with daily cigarette consumption after adjusting for age, BMI, and alcohol intake (α = 1.040, P = 0.001, Figure 2C), though this association was observed only in the youngest of the four age subgroups (α = 1.108, P < 0.001).

SHBG was significantly higher among current smokers who smoked > 20 cigarettes/day (44.52 nM) than among those who smoked 10-20 cigarettes/day (42.78 nM, P = 0.008) or < 10 cigarettes/day (41.64 nM, P < 0.001, Figure 2D). SHBG was positively associated with daily cigarette consumption after adjusting for age, BMI, and alcohol intake (α = 1.029, P = 0.001, Figure 2D), and this association was observed in the 40-49 (α = 1.070, P < 0.001) and 50-59 (α = 1.033, P = 0.028) age subgroups.

### Associations between cigarette smoking and AMS score

### Smoking status

**Figure 3:**
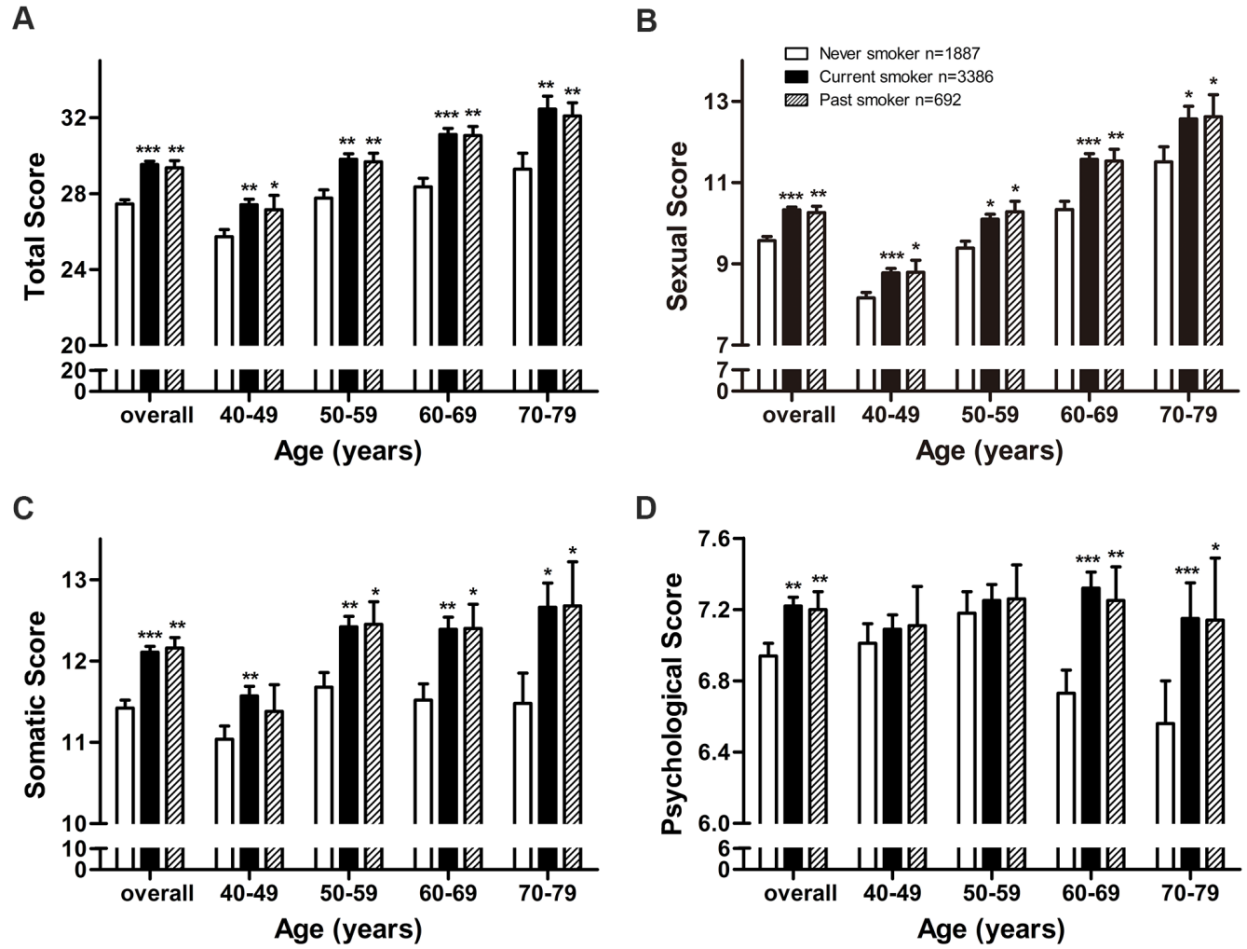
**Adjusted mean AMS scores by smoking status in middle-aged and elderly men.** Adjusted mean (**A**) total score, (**B**) sexual score, (**C**) somatic score, and (**D**) psychological score by smoking status in middle-aged and elderly men. Arithmetic mean values were calculated using multiple covariance and adjusting for age (in “overall” analysis only), body mass index, and alcohol intake. Error bars indicate standard error. *P < 0.05, ** P < 0.01, *** P < 0.001 vs. never smokers.

Total AMS score was significantly higher among current smokers (29.53, P < 0.001) and past smokers (29.36, P = 0.003) than among never smokers (27.45, Figure 3A), and higher scores for current smokers were observed in all four age subgroups. Similar results were observed for sexual score (Figure 3B) and somatic score (Figure 3C).

Psychological score was significantly higher in current smokers (7.22, P = 0.004) and past smokers (7.20, P = 0.019) than in never smokers (6.94, Figure 3D), and these results were observed in the two older age subgroups.

### Number of cigarettes smoked per day

**Figure 4:**
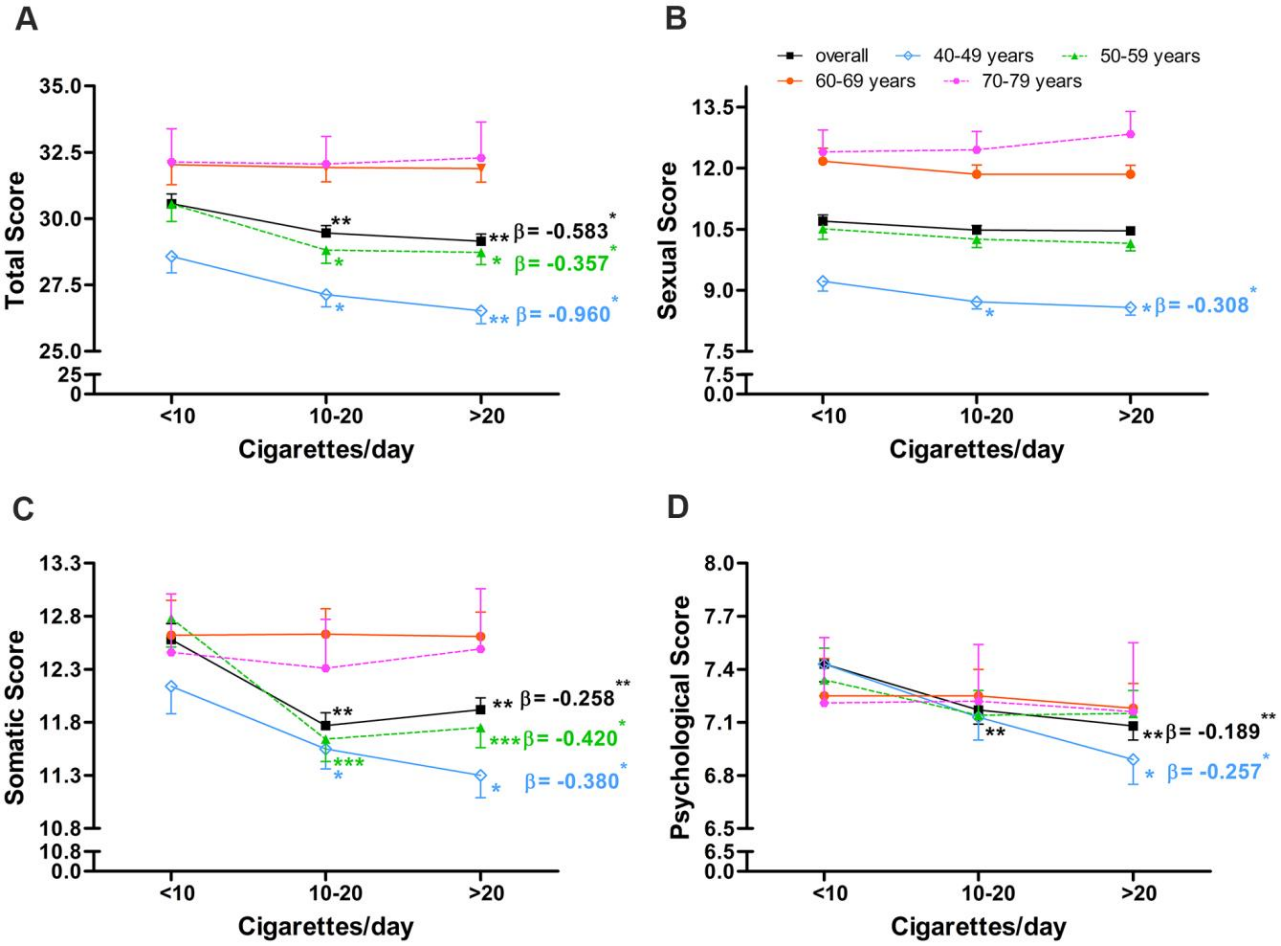
**Association between number of cigarettes smoked per day and AMS scores in middle-aged and elderly men.** Association between number of cigarettes smoked per day and (**A**) total score, (**B**) sexual score, (**C**) somatic score, and (**D**) psychological score in middle-aged and elderly men. Arithmetic mean values were calculated using multiple covariance and adjusting for age (in “overall” analysis only), body mass index, and alcohol intake. Error bars indicate standard error. *P < 0.05, ** P < 0.01, *** P < 0.001 vs. current smokers who smoked <10 cigarettes/day. Regression coefficients were obtained via multiple regression adjusting for age (in “overall” analysis only), body mass index, and alcohol intake and were calculated separately for daily cigarette consumption subgroups, which were ordinally categorized as <10, 10-20, >20 cigarettes/day. *P < 0.05 and ** P < 0.01 for regression coefficients.

Among current smokers, total AMS score was significantly lower among those who smoked > 20 cigarettes/day (29.15, P = 0.002) and 10-20 cigarettes/day (29.46, P = 0.001) than among those who smoked < 10 cigarettes/day (30.56, Figure 4A). Total score was negatively associated with daily cigarette consumption after adjusting for age, BMI, and alcohol intake (α = -0.583, P = 0.011, Figure 4A), and this negative association was observed in the two youngest age subgroups (40-49: α = -0.960, P = 0.015; 50-59: α = -0.357, P = 0.023). Similar results were observed for somatic score (Figure 4C). Similar results were also observed for psychological score, except that the negative association was observed only in the 40-49 age subgroup (Figure 4D).

Sexual score did not differ significantly among the three daily cigarette consumption subgroups, and no association was observed between sexual score and daily cigarette consumption after adjusting for age, BMI, and alcohol intake (Figure 4B). Nevertheless, a negative association was observed in the 40-49 age subgroup (α = -0.308, P = 0.041).

## DISCUSSION

This is the first large-scale cross-sectional study of associations between cigarette smoking, sex hormone levels, and LOH symptoms in middle-aged and elderly men. We found that TT, CFT, LH, and SHBG levels, as well as sexual, somatic, psychological, and total AMS scores, were higher in current smokers than in never smokers. These associations depended on age: as men aged, the positive associations between cigarette smoking and TT, CFT, SHBG, and LH levels weakened, whereas the positive association between smoking and AMS scores became stronger. The positive associations between smoking and sex hormone levels and AMS scores suggest that smoking may help explain why testosterone deficiency and LOH symptoms are seldom observed in the same individual and, therefore, why LOH can be so challenging to diagnose [1, 2, 4, 28]. In addition, while TT, CFT, LH, and SHBG levels were similar in past smokers and never smokers, AMS scores in the sexual, somatic, and psychological dimensions were significantly higher in past smokers than in never smokers. This suggests that sex hormone levels, but not LOH symptoms, can revert after smoking cessation.

Few studies have assessed interactions between smoking history and age on sex hormone levels in men. Although several studies have demonstrated associations between smoking and higher TT and SHBG levels [20, 21, 27], associations between smoking and free testosterone and LH levels remain controversial [20, 21, 25, 26]. In the present study, smoking was associated with higher TT and SHBG levels over a wide age range, whereas the positive associations between smoking and CFT and LH were observed over a much narrower age range; this may help explain the inconsistent findings of previous studies.

Although the biological basis for positive associations between cigarette smoking and testosterone levels remains unclear, a smoking-related increase in SHBG, which can inhibit testosterone degradation, might be involved [29, 30]. Consistent with this hypothesis, we observed concurrent increases in TT and SHBG among current smokers. The mechanisms underlying the positive association between smoking and SHBG remain unclear. Further studies are needed to identify those mechanisms as well as which of the more than 4000 chemicals in cigarettes are involved in the smoking-induced stimulation of SHBG. Regulation of SHBG is complex, and many factors can promote its activity, including estrogen, thyroid activity, low BMI, TNF-α, exercise, and a vegetarian diet, among others [31, 32]. In contrast, some of the factors that inhibit SHGB include androgens, prolactin, insulin, insulin-like growth factor-1, body fat, and a fatty diet [31, 32]. In the present study, higher levels of both testosterone and SHBG were observed in current smokers less than 70 years old, suggesting that increased SHBG activity in current smokers is not secondary to variations in serum testosterone associated with smoking. Piontek et al. reported that the cigarette metabolites trigonelline and 4-ethylphenylsulfate were associated with serum SHBG levels [33]. Smoking also increased serum TNF-αlevels and decreased appetite, body fat, and BMI [34–36], which might help account for higher SHBG levels in current smokers.

The higher testosterone levels observed in this study among current smokers 40-49 years old might also be a result of the concurrent increase in LH levels observed in the same group. Although the mechanisms underlying associations between smoking and LH levels, which can affect the hypothalamus-pituitary system in multiple ways, have not been fully characterized, nicotine plays a major role. According to studies in male non-smokers and rats, nicotine activates cholinergic receptors in dopamine, γ-amino butyric acid, and opioid peptide neurons in the hypothalamus, stimulating the release of the corresponding neurotransmitters [37–39]. These neurotransmitters can inhibit the activity of gonadotrophin-releasing hormone (GnRH) pulse generator neurons in the medial eminence of hypothalamus, which in turn inhibits pulsatile LH secretion from the pituitary [37–39]. However, in contrast to non-smokers, no inhibition of pulsatile LH secretion after nicotine exposure was observed in male current smokers [39]. This suggests that smokers can develop compensatory mechanisms to counteract the inhibitory effect of nicotine on pulsatile GnRH and LH secretion [39]. On the other hand, nicotine can activate noradrenaline neurons in the median eminence of the hypothalamus via the cholinergic receptor, which increases noradrenaline release and turnover [40]. Increased noradrenaline activity can stimulate GnRH secretion and subsequent pulsatile LH secretion from the pituitary [40]. In addition, nicotine can immediately bind to and activate GnRH neurons in the medial preoptic nucleus of hypothalamus and subsequently stimulate the pulsatile secretion of GnRH and LH [41]. In summary, nicotine affects GnRH secretion via multiple complex and inter-related systems which include both inhibitory and stimulatory mechanisms, and current smokers can develop tolerance to many of these effects [39]. The overall effect of nicotine on GnRH and LH secretion in current smokers may therefore depend on additive effects of two opposing systems. In the present study, LH levels were higher in current smokers aged 40-49 years than never smokers, suggesting that the stimulatory effects of nicotine were stronger than its inhibitory effect in that group. However, our results indicated that current smokers more than 49 years old had developed a tolerance to those stimulatory effects.

No positive associations between smoking and LH and SHBG levels were observed in men older than 50 or 70 years, respectively, which may reflect a weaker response by the pituitary gland and liver to cigarette smoking with aging. In addition, the similar sex hormone levels observed here between past smokers and never smokers across all age subgroups suggests that the pituitary gland and liver can restore appropriate levels of sex hormones after smoking cessation.

Higher levels of TT and CFT, which were observed in current smokers less than 70 and 60 years old, respectively, are generally thought to be negatively associated with LOH symptoms [1, 4, 28]. However, we found that sexual, somatic, and total AMS scores were higher in current smokers aged 40-79 years despite higher levels of TT and CFT. This may reflect the development of smoking-induced LOH symptoms which overpowers any protective effects of higher TT and CFT levels [11–19]. This hypothesis should be explored in detail, especially given our potentially surprising finding that scores for all three AMS domains in current smokers 40-49 years old and for somatic AMS scores in current smokers aged 50-59 years old decreased as daily cigarette consumption increased. It is possible that higher testosterone levels in current smokers may help protect against LOH; daily cigarette consumption was positively associated with serum TT levels and negatively associated with AMS scores in current smokers 40-59 years old, and daily cigarette consumption was no longer associated with serum TT or AMS scores in current smokers older than 60 years. Nevertheless, the hazards of smoking appear to outweigh the benefits, and current smokers presented worse LOH symptoms than never smokers. Together, these findings suggest that andrologists should exercise caution when evaluating associations between LOH symptoms with hypogonadism in male patients, and that smoking status should be considered as a confounding factor. In addition, AMS scores were higher in past smokers than in never smokers, which suggests that smoking contributes irreversibly to the development of LOH symptoms.

Both LOH-related symptoms and testosterone deficiency are required for the diagnosis of LOH. However, a portion of symptomatic men have normal serum TT levels [1]. The reason for this paradox remains unclear. Sandro et al. suggested that a higher than expected annual testosterone decrease velocity might contribute to this phenomenon; if serum TT levels decrease to a greater extent in a particular individual compared to the decrease observed in healthy men at the same age, he tends to develop LOH-related symptoms even if his serum TT level is in the normal range [42]. Our results provide another possible explanation for this paradox; smoking, which is positively associated with sex hormone levels and AMS scores in middle-aged and elderly men, may contribute to LOH-related symptoms in men with normal serum TT levels.

This may be the first study to report that smoking is positively associated with sex hormone levels and AMS scores in middle-aged and elderly men. Furthermore, our results indicate that the effects of smoking on sex hormone levels and LOH symptoms vary with age. These findings, if verified in longitudinal studies, may help explain why LOH can be difficult to diagnose and should be incorporated into diagnostic guidelines. Notably, men with diseases affecting the hypothalamus-pituitary-testis axis were not included in this study, and caution should be taken when extending the conclusions to such patients, e.g., those with Klinefelter syndrome [43].

## MATERIALS AND METHODS

### Study subjects

This multi-center, cross-sectional study was conducted between June 1, 2013 and August 31, 2016 in six provinces in China (Jiangsu, Guizhou, Shanxi, Hebei, Guangdong, and Hubei) that were selected based on logistical support and to ensure a mixture of geography, socioeconomic status, and lifestyle representative of the entire country. Stratified, random-cluster sampling was performed. First, the population in each province was stratified into 5 layers according to the administrative level, and one community (urban or rural) was sampled for each layer. Second, random-cluster sampling was used to select 1 community in each layer of the province. Finally, 5 communities (urban or rural) were sampled for each province.

All men aged 40-79 years in the selected communities were invited to participate. Subjects were excluded from the study if they had any disease of the hypothalamus-pituitary-testis axis, including primary sterility, congenital hypogonadism, Kallmann syndrome, hypothalamus glioma, hypophysoma, germinoma, or orchitis. Subjects were also excluded if, during the previous three months, they had taken any of the following medications that may affect testosterone level: glucocorticoid, sex hormones, thyroid hormones, insulin, growth hormone, anti-prostatic hyperplasia drugs, or anti-tumor drugs. The study was conducted in accordance with the Declaration of Helsinki, and the protocol was approved by the Ethics Committee Review Board of Tongji Medical College of Huazhong University of Science and Technology. All participants signed informed consent forms.

### Assessment of smoking status and LOH symptoms

Information on demographic characteristics (age, race/ethnicity, education, residence, occupation, marital status), cigarette smoking (smoking status, smoking years, and number of cigarettes smoked per day), and alcohol intake were collected with a structured questionnaire that was completed by the subject. According to smoking status, participants were categorized as never smoker, current smoker (men who had smoked cigarettes for ≥6 months and were still smoking at the time of interview), or past smoker (men who had stopped smoking at least 6 months before the interview) [21]. Based on the number of cigarettes smoked per day, current smokers were further categorized as mild (<10), moderate (11-20), or heavy smokers (>20). A validated Chinese version of the AMS scale [7] was used to assess LOH symptoms. Data on medical conditions, medications, and LOH symptoms were collected with the assistance of an andrology clinician during a face-to-face interview.

### Sex hormone assays and BMI measurement

Height and weight were measured before the face-to-face interview, and BMI was calculated as mass (kg)/height (m)^2^ [44]. A fasting venous blood sample was collected between 7:00 a.m. and 11:00 a.m., and serum was isolated, stored at -70° C, and assayed for TT, LH, and SHBG using standard chemiluminescent immunoassays (Beckman Coulter, USA) on a Beckman Access Immunoassay system (Beckman Coulter, USA). Between-day coefficients of variation for TT were 8.10% at 0.35 ng/mL and 6.26% at 12.88 ng/mL; SHBG, 5.4% at 6.3 nM and 5.2% at 171 nM; and LH, 6.4% at 4.01 IU/L and 5.4% at 55.04 IU/L. Serum calculated free testosterone (CFT) was calculated based on TT and SHBG as described [45].

### Statistical analysis

All statistical analyses were performed using SPSS 25.0 (IBM, Chicago, IL, USA). Categorical variables are expressed as number and percentage (%), and inter-group differences were assessed for significance using the chi-squared test. Normally distributed continuous variables are expressed as mean ± standard error (SE), and inter-group differences were assessed using analysis of covariance followed by the Student-Newman-Keuls test for pairwise comparisons. Levels of sex hormones and SHBG were positively skewed, so a natural logarithm transformation was applied prior to analysis. Adjusted mean sex hormone levels and AMS scores were calculated by adjusting for age, BMI, and alcohol intake; these means were compared using analysis of multiple covariance followed by the Least-Significant-Difference test for pairwise comparisons. Multiple linear regression was used to examine whether cigarette consumption showed a dose-response relationship with sex hormone levels or AMS scores. Additional subgroup analyses were performed after stratifying subjects by age in 10-year increments (40-49, 50-59, 60-69, or 70-79 years). All *P* values were 2 sided and *P* < 0.05 was considered significant.
